# Using nudging and social marketing techniques to create healthy worksite cafeterias in the Netherlands: intervention development and study design

**DOI:** 10.1186/s12889-016-3927-7

**Published:** 2017-01-11

**Authors:** Elizabeth Velema, Ellis L. Vyth, Ingrid H. M. Steenhuis

**Affiliations:** Department of Health Sciences and the EMGO+ Institute for Health and Care Research, Faculty of Earth and Life Sciences, Vrije Universiteit Amsterdam, De Boelelaan 1085, 1081 HV Amsterdam, The Netherlands

**Keywords:** Nudging, Social marketing, Worksite cafeteria, Purchasing behavior, Employee, Overweight, Randomized controlled trial

## Abstract

**Background:**

The worksite cafeteria is a suitable setting for interventions focusing on changing eating behavior, because a lot of employees visit the worksite cafeteria regularly and a variety of interventions could be implemented there.

The aim of this paper is to describe the intervention development and design of the evaluation of an intervention to make the purchase behavior of employees in the worksite cafeteria healthier. The developed intervention called “the worksite cafeteria 2.0” consists of a set of 19 strategies based on theory of nudging and social marketing (marketing mix). The intervention will be evaluated in a real-life setting, that is Dutch worksite cafeterias of different companies and with a number of contract catering organizations.

**Methods/design:**

The study is a randomized controlled trial (RCT), with 34 Dutch worksite cafeterias randomly allocated to the 12-week intervention or to the control group. Primary outcomes are sales data of selected products groups like sandwiches, salads, snacks and bread topping. Secondary outcomes are satisfaction of employees with the cafeteria and vitality.

**Discussion:**

When executed, the described RCT will provide better knowledge in the effect of the intervention ﻿“the worksite cafeteria 2.0” on the purchasing behavior of Dutch employees in worksite cafeterias.

**Trial registration:**

Dutch Trial register: NTR5372.

## Background

### Introduction

Rates of overweight in the Netherlands are high. To illustrate, in 2014, 43% of Dutch men and 31% of Dutch women were overweight [1]. Overweight is associated with the incidence of co-morbidity such as type II diabetes, cardiovascular diseases and several types of cancer [2] which underpins the importance of targeting this health problem. Additional to the burden of disease, also healthcare spending and costs of sick leave stress the concern of the increasing prevalence of overweight and obesity [3–6].

Overweight and obesity are generally the result of an imbalance between energy intake (eating) and energy expenditure (physical activity) [7]. The current “obesogenicity” of the environment, which means an abundant availability, easy accessibility and aggressive marketing of foods, together with declines in physical activity, makes it difficult not to gain weight [8].

A commonly used strategy in decreasing overweight is to focus on changing eating behaviors. Eating behaviors influence energy intake through choices about when and where to eat, and the types and amounts of foods chosen, including decisions about starting and stop eating [9, 10]. Moreover, interventions with a dietary component result in weight loss [11]. A suitable location for targeting eating behavior could be the worksite cafeteria, since it is a natural social context where most employees eat at least one meal during their workday. The Netherlands has a working population of more than 7 million people [12] of which about 45% have lunch daily at the worksite cafeteria [13]. Thereby, choosing the worksite cafeteria as a location to intervene in eating behavior gives the opportunity to reach people more than once as they visit the worksite cafeteria regularly. Finally, worksites could potentially reach a large part of the adult population including many who have not traditionally been engaged in health promotion activities [14, 15].

Regarding the dietary intake of employees, improvements can be made. Although little is known about the current health status of Dutch worksite cafeterias, several studies show adverse effects of (associations with) foods produced and eaten outside the home. For instance, out-of-home eating has been associated with a higher energy and fat intake [16, 17], a higher energy density [18] and food portions in places to eat outside the home exceed standard portion sizes [19]. Large portions in turn have been related to a higher energy intake [20–23].

Today Dutch worksites cafeterias have already been used as a setting for interventions focusing on changing eating behavior [24–29]. For example, the placing of informational sheets near food products with the caloric (kcal) value of a product translated into the number of minutes to perform a certain (occupational) activity [24], or the labeling of low-fat products [26]. Results of these interventions however were mixed. The environmental intervention of Engbers et al. [24], was modestly effective in changing behavioral determinants towards eating less fat (social support, self-efficacy and attitude), but ineffective in positively changing actual fat, fruit and vegetable intake of office workers. Labeling low-fat products also showed partial effectiveness. For the whole study population no significant effects on consumption data were found. The data however did show a beneficial and significant treatment effect of the labeling program on total fat intake for respondents who believed they ate a high-fat diet. Sales data revealed a significant effect of the labeling program on desserts, but not for the other products [26].

Also outside the Netherlands strategies to improve eating behavior in the worksite cafeteria are studied. For instance, increasing the availability of healthy foods like fruits and vegetables and products low in energy density [30, 31], offering smaller portions [32], providing nutrition information on menus [33, 34] placing a sign with the message “Pick me! I am low calorie” on the low-fat milk [35], or showing a nutrition logo on healthy products [15].

However, not all strategies are effective in improving eating behavior [29] and the quality and reporting of worksite intervention studies is low [36], so searching for a new approach is needed.

A method introduced in this setting recently is the concept of nudging [37]. Nudging is defined as changing the presentation of choice options in a way that it makes the desired choice – in our case the healthier option - the easy, automatic and default option, without forbidding any options [38]. Nudges can be seen as relatively simple, easy to implement and inexpensive interventions. Besides, consumers preservation of liberty of choice is a key characteristic of nudging [38]. Another strength of this relatively new strategy is the fact that it is effortless for consumers because it does not result in ego depletion [39]. Ego depletion is the phenomenon that acts of self-control at Time 1 reduce performance on subsequent, seemingly unrelated self-control tasks at Time 2 [40]. In this new field of nudging strategies, the focus is most often on the effect of one or two strategies within one intervention, for instance, Van Kleef et al., [41] tested the nudge of offering healthy snacks in larger shares and at higher shelves at the checkout counter in a hospital staff restaurant. However, the character of nudges, not depleting self-control, make them suitable to use simultaneously. A combination of mostly proven effective nudging strategies would have potential to result in a cumulative effect, and has to our knowledge never been studied before, especially not in worksite cafeterias.

Next to nudging also relatively new in the field of intervention development for health promotion is social marketing. Social marketing seeks to develop and integrate marketing concepts with other approaches to influence behaviours that benefit individuals and communities for the greater social good [42]. Furthermore, social marketing aims to change behaviour, by getting acquainted with the target audience. Social marketing is considered a useful tool in changing peoples’ health behaviour. Stead et al., [43] found in their review that there was evidence that interventions adopting social marketing principles could be effective across a range of behaviours, with a range of target groups, in different settings, and can influence policy and professional practice as well as individuals [43]. Carins et al., [44] who also conducted a review, stated that social marketing when employed to its full extent offers the potential to improve healthy eating behavior [44].

Some social marketing strategies can be seen as a form of nudging. They aim to change behavior and do not forbid undesirable behavior. Shaping the food environment by the use of nudging and social marketing techniques seems a promising strategy to examine in order to change purchasing and subsequently eating behavior. The worksite cafeteria is a suitable food environment to shape.

Considering this, the objective of this study is to develop an intervention, named: “the worksite cafeteria 2.0”, based on nudging and social marketing techniques to improve eating behavior of Dutch employees. Subsequently the aim is to describe the design of a study to measure the effect of multiple simultaneously executed strategies in “the worksite cafeteria 2.0” on purchasing behavior of visitors in Dutch worksite cafeterias. The research question of the described study protocol will be; What is the effect of a healthier worksite cafeteria based on nudging and social marketing techniques on the purchasing behavior of employees?

## Methods and research design

### Design

**Fig. 1:**
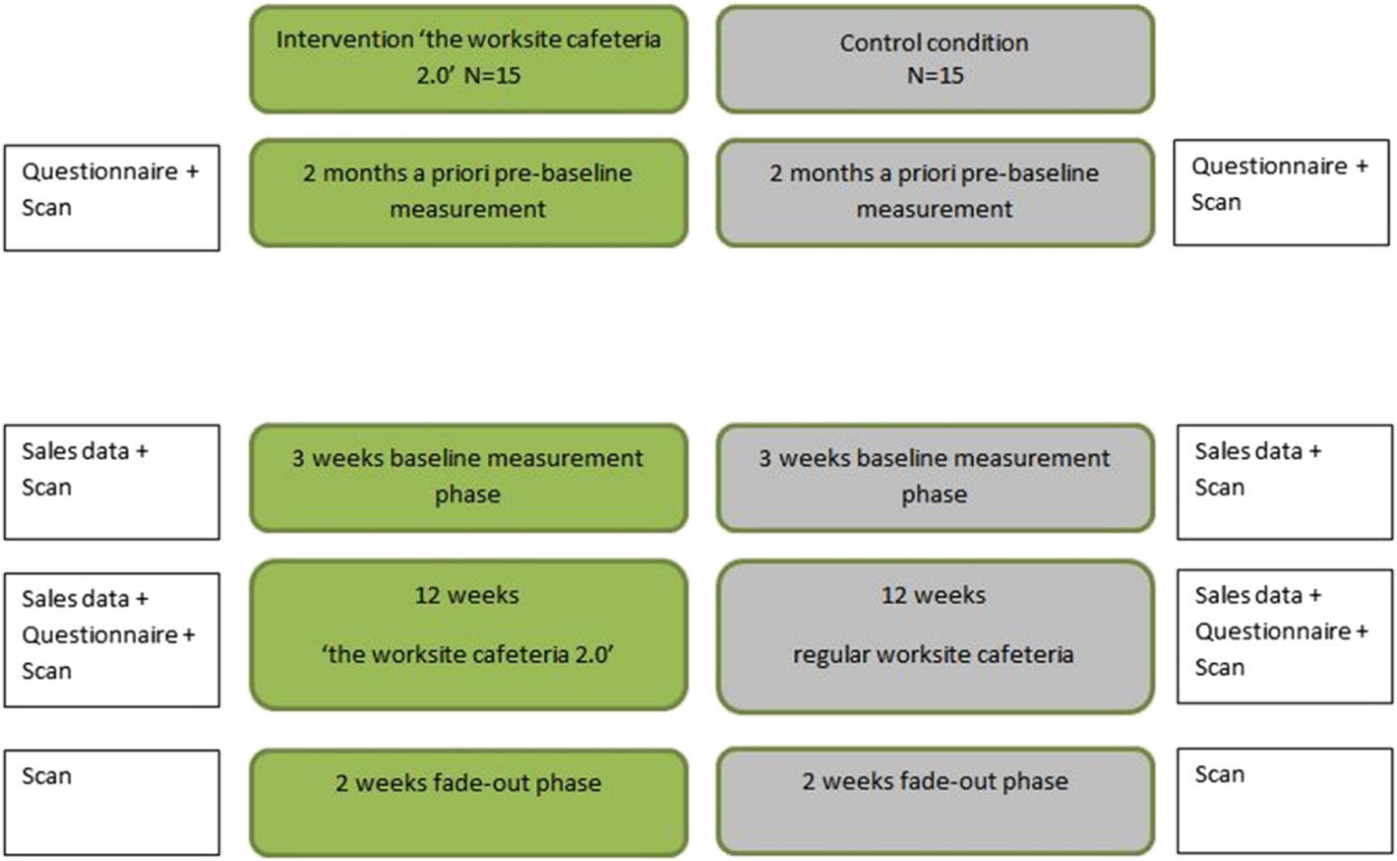
Time planning of measurements RCT

The effects of a healthier worksite cafeteria will be studied by means of a two-arm, (pre-stratified) randomized controlled trial (RCT). The RCT is designed to evaluate the effect of a 12-week intervention in the worksite cafeteria that is aimed at changing food choices in the worksite cafeteria towards healthier ones. A linear mixed model is used to also execute repeated measures. Primary outcomes are sales data of products in eight product groups, measured via cask register output. Secondary outcomes include satisfaction with the worksite cafeteria and vitality. The sample will include approximately 34 worksite cafeterias of 6 different catering companies. Worksite cafeterias will be randomly assigned (1:1) to the intervention or control arm. The randomization will be a block randomization with the size of worksite cafeterias (<500 or ≥500 customers daily) and order of inclusion as blocking variables, performed by the researcher. Outcome measures will be collected at baseline and weekly during the 12-week intervention phase to assess changes in food choice behavior of visitors. Figure 1 provides an overview of the timeline of the study design. The Medical Ethics Committee of the VU Medical Centre confirmed that this study does not apply to the Medical Research Involving Human Subject Act (WMO), due to the nature of the measurements (sales data and anonymous questionnaires).

### Intervention

The intervention called “the worksite cafeteria 2.0” consists out of 19 strategies (Table 1), all with a probability to result in healthier food behavior. The strategies are divided over four elements; the so-called 4P’s from marketing: Product, Place, Price and Promotion.

**Table 1:** Intervention strategies and references

	Strategie	Reference
	Product	
1	In every product category at least 1 product of better choice is visibly offered.	[56]
2	A warm lunch meal is also offered in a smaller portion.	[27]
3	Fruit and vegetables are offered.	[30]
4	Fruit and vegetables are offered ready to eat (peeled).	[57, 58]
5	Water is offered for free.	[59]
6a	The visible share of healthy (better choice) products is at least 60%.	[60]
6b	The visible share of healthy (better choice) products is at least 80%.	[60]
7a	Warm snacks^a^ are offered up to three days a week.	[61]
7b	Warm snacks^a^ are offered up to one day a week.	[61]
8	Salads are offered without dressing and with different vegetables.	[48, 62]
	Place	
9	Healthy products are in the beginning of the route. These products are: salads, fruit & vegetables, bread, bread topping and healthy sandwiches^b,c^.	[46]
10	Of every product group the preferred product or presentation of this product is most visible (at front on eye level).	[54, 63]
11	In case of a shelf at the cash desk it is partly filled with fruit & vegetables. Fruit & vegetables are on top or at front.	[41]
11a	In case of a shelf at the cash desk it is only filled with fruit & vegetables.	[41]
	Price	
12	A relatively cheap combi-deal is offered with milk^d^/coffee/tea/vegetable juice, sandwich^b,c^, and fruit with a price comparable with the average price of a sandwich in the same restaurant.	[64]
13	Prices of warm snacks^a^ (e.g. chicken nuggets) are 25% increased and prices of healthy sandwiches^b, c^ are 25% decreased.	[65–67]
14	Within a product category preferred products are 25% lowered in price and exception products are 25% higher in price compared with the normal prices in same restaurant.	[65–67]
	Promotion	
15	There is only promotion of food products in the preferred category (or the Choice criteria for combined meals).	
16	When a healthy product is promoted is has a recognizable, permanent spot in the restaurant.	
17	On the menu, e.g. on displays or intranet the healthy products are named first.	[68]
18	On the menu healthy dishes are presented in an attractive way.	[69]
19	Healthy products are promoted with temporary campaigns like with a stand.	

^a^Snacks contain all fried snacks like fries, chicken nuggets, or spring rolls, but also puff pastry snacks like sausage rolls and cheese rolls

^b^‘Healthy’ sandwiches that meet the criteria of the Choice logo

^c^This can also be a salad that meets the criteria of the Choice logo. In collaboration with dietitians of all catering companies a list with products will be formed

^d^This can also be buttermilk or a semi-skimmed milk drink without added sugar

“The worksite cafeteria 2.0” is developed based on nudging and social marketing strategies and corresponds with the Guidelines Healthy Canteens [45]. The guidelines for healthy canteens are developed by the Netherlands Nutrition Centre in collaboration with scientific experts on food and behavior and users of these guidelines like caterers. The guidelines offer strategies about how to arrange a sport or school canteen or worksite cafeteria that induces visitors to show healthier eating behavior. We developed the intervention in four phases: collecting strategies from literature, qualitative face to face expert interviews, qualitative focus group interviews with employees of different Dutch companies and a feasibility pilot study. The first phase consisted of deriving effective strategies from the field of food behavior and marketing science (e.g. serving healthy foods first in buffet lines improves overall meal selection [46]). Second, experts in the field of contract catering, nutrition and facility management were consulted to identify promising strategies within current effective strategies, taking the feasibility in catering practice and their expertise into account. This was done be conducting eight semi-structured interviews with fourteen experts (publication in preparation). Third, the views and motivations of the target population, namely Dutch employees who regularly visit a worksite cafeteria, towards choosing lunch were obtained. Therefore seven focus group interviews, with 45 employees, were conducted (publication in preparation).

The fourth phase consisted of a feasibility pilot study in two worksite cafeterias in order to test the feasibility of the intervention strategies (not published).

### Sample sizes

The power calculation is based on the main outcome measure of the linear mixed model: sales data of sandwiches, sandwich filling, salads, (hot) meals, fruit and vegetables, ‘combi-deals’, snacks and candy. Using a standard deviation of 10%, a sample of 15 intervention and 15 control worksite cafeterias are needed to detect a 20% mean increase in ‘better choice’ products between the intervention and the control group, at 80% power, a 5% level of significance and an estimated intra-cluster correlation (ICC) coefficient of sales within worksites of 0.15. The ICC represents how strongly sales in one worksite cafeteria are related. This increase of 20% is based on the sales of sandwiches and snacks in a pilot study testing this intervention (not published). The standard deviation of 10% is based on the same pilot study. To account for a possible 10% drop out of location or sudden difficulties like incorrect cash desk registration, 34 worksite restaurants will be randomized [26, 27, 29] and divided over the experimental group and the control group. By comparisons of the sales data between the experimental and the control group the effect of the healthy worksite cafeteria strategies can be studied.

### Recruitment of worksite cafeterias

Thirty-four worksite cafeterias will be recruited to participate in the study. All caterers who are a member of the trade association for Dutch catering companies ‘Veneca’ are asked to provide worksites from some of their clients (companies) to join in the study. The three biggest catering companies affiliated with Veneca have a market share of 80% in of the Dutch contract catering market. Recruitment of worksite cafeterias will be done in different ways. The catering companies will be approached by the Quality Committee of Veneca. The Quality Committee consists of representatives of all members of Veneca. They are concerned with topics like sustainability and health in contract catering. By means of multiple presentations from the researcher for the Quality Committee and supplementary letters for recruitment, caterers are being able to inform their customers about joining in the study. Also catering companies not being member of Veneca will be encouraged to join. This will be done by means of promoting the study on a national human resource congress, a call at an online radio station (werken.fm), an article in a magazine for the hotel and catering industry, in a national newspaper and by informing the sustainability working group of government agencies about the study. In order to ensure the representativeness of worksite cafeterias caterers will be encouraged to approach clients of different types of businesses, like factories. The researchers will decide whether the worksite cafeterias comply with the inclusion criteria.

### Inclusion criteria

Inclusion criteria for worksite cafeterias are 1) a minimum of 100 lunch customers per day, to ensure sufficient sales, 2) a cash desk system that can register separate products, in order to measure sales shifts within products groups, 3) cash desks are staffed or all products must be scanned, to ensure accurate registration, 4) the worksite cafeteria or the company will not organize active nutritional or health campaigns from January 2016 until August 2016, because it could interfere with the effect of the intervention, 5) the company gives permission to change the selection of products for 12 weeks during the experiment, 6) the company gives permission to change the routing in the restaurant for 12 weeks during the experiment, 7) the company gives permission to change the price of products for 12 weeks during the experiment, 8) the company gives permission to change the promotion of products and menu for 12 weeks during the experiment, 9) the company gives permission for measuring sales data during the study, 10) the company gives permission for conducting a questionnaire within their employees. To finalize the inclusion the researcher, together with the account manager of the catering company, will visit the worksite cafeteria for a meeting with the employer or his representative, to make sure all conditions for participating in the research are clear.

### Implementation

After conducting the randomization, all catering teams of the intervention worksite cafeterias will be visited by the researcher and their usual account manager from the catering company. In this meeting the researcher will explain all strategies and train the cafeteria managers to instruct their team. In the phase between randomization and start of the intervention, several training sessions will be planned with the catering manager and the researcher.

### Measures

This project will use three ways of data collection: sales data, a worksite cafeteria scan and a questionnaire. All measures are quantitative and will be done the same way in both intervention and control worksites. Sales data are the primary outcome measure and will be objectively measured by obtaining cash register output. The worksite cafeteria scan, from here referred to as ‘scan’ is a checklist to objectively measure the degree in which the intervention is executed correctly, or in the case of the control group, the extent to which the worksite cafeteria already applies strategies that are also part of the bundle of strategies from the intervention “the worksite cafeteria 2.0”.

The questionnaire will obtain subjective data of the employees visiting the worksite cafeteria. Employees of all participating companies (both experimental and control group) will fill in the questionnaire at the pre-measuring phase and during the intervention phase.

Figure 1 shows all measures within the time frame.

### Sales

Daily sales of sandwiches, sandwich filling, salads, (hot) meals, fruit and vegetables, ‘combi-deals’, snacks and candy will be registered for 15 weeks (3 weeks pre-measuring and 12 weeks intervention) in both intervention and control group worksite cafeterias.

All food products can be classified for relative healthiness, in one out of three categories within its product group. The classification is based on the levels of saturated fat and trans fat, added sugar, salt, dietary fiber and overall energy density [47–49]. Products can be classified in the following categories; the ‘preference category’, which is the most healthy category, the ‘middle category’ which is less healthy, but still reasonable, or the ‘exception category’, for products most unfavorable within the product category. The first two categories ‘preference category’ and ‘middle category’ are taken together into the so-called ‘better choice’. This provides a dichotomy within product groups; ‘better choice’ products, versus ‘exception’ products [48].

The primary outcome measure of this research project is the proportion of sales of ‘better choice’ products within the product categories sandwiches, sandwich filling, salads, (hot) meals and snacks and the sales of fruit and vegetables, ‘combi-deals’ and candy. The difference in (proportions of) sales of these products will be compared between the intervention group and the control group. All measured product categories correspond to the intervention strategies. In Dutch worksite cafeterias prepared sandwiches, bread combined with separate toppings or fillings and snacks are common lunch items [50], therefore certain intervention strategies target these products. The sales data will provide insight in the effect of the larger visible share, better pricing, placement and promotion of healthier ‘better choice’ products and the effects of not promoting less healthy products like snacks.

### Worksite cafeteria Scan

The worksite cafeteria scan (scan) is a measuring tool to scan the degree of implementation of the intervention. For all strategies in the intervention it is measured to what extend they are executed correctly. The scan consists of a checklist with all the 19 strategies in the intervention. For each strategy has to be scored if it is executed and how it is executed (‘correct’ or ‘incorrect’). The scan is not tested for reliability and validity, however the researcher who will train the worksite cafeteria managers on how to execute the strategies, is the same to scan the degree of use and implementation of the strategies before and during the intervention phase. Also worksite cafeterias in the control group will be scanned to be able to compare their status with the intervention cafeterias. Both the researcher and one trained research assistant will execute the first scan in a worksite cafeteria together, to take care of the validity. When no discrepancies occur, both researchers will perform scans on their own. During the 12-week intervention phase, bi-weekly scans are executed unannounced in the intervention cafeterias. The control restaurants will be scanned every four weeks by the researcher or research assistant.

### Questionnaires

To gain insights into the satisfaction of guests about the worksite cafeterias, employees of all worksites will be asked to fill in an online questionnaire at baseline and after the intervention phase. The questionnaire assesses elements of the satisfaction with the worksite cafeteria and vitality with the Vita-16 [51]. Further, self-reported demographic variables will be collected like age, sex, body weight, height, level of education, marital status, household size, frequency of having lunch at the worksite cafeteria and the proportion of lunch purchased in the worksite cafeteria. Concepts like frequency of having lunch at the worksite cafeteria were tested by two researchers (IS and ELV). They tested if the answer categories were appropriate and if questions were stated clear and neutrally. A small test panel of eight persons tested the questionnaire thereafter. They reviewed the questionnaire on clarity and gave feedback. The feedback was used to improve the questionnaire.

Also demographic characteristics of the companies will be measured by the researchers, like work sector (white collar, blue collar) and size of the company (amount of employees). Worksite cafeterias demographic and geographic characteristics that are measured are size (visitors daily), area (urban, suburban or rural), amount and proximity of competing lunch venues/purchase points for food, catering company (name, size and formula), contract form and mean amount of money spent per visitor per lunch.

### Statistical analysis sales data

We will use a linear mixed model (LMM) analysis to compare the intervention and control group. We distinguish three levels of data: time (level 1), the individual worksite cafeteria (level 2) and the catering companies (level 3). We adjust for this clustering of our data via a linear mixed model, including random intercepts and slopes where necessary according to the common procedure described in Twisk [52].

### Statistical analysis Worksite cafeteria Scan

The worksite cafeteria scan is an instrument to measure to which level the intervention is executed and if it is executed correctly. For each strategy can be filled out if this is executed (yes/no) and if it is correctly or incorrectly conducted. A percentage of correctly implemented strategies will be the result of the scan. Strategies that are not applicable will kept out the calculation.

We will not test for baseline differences based on arguments of De Boer et al. [53] to actively adopt the CONSORT 2010 statement by not publishing significance tests for baseline differences. Adjustment for prognostic variables will nevertheless be made. We will report results from the fully adjusted as well as crude analyses.

Also the level of correctly executing the intervention will be measured in the intervention group during the intervention phase (12 weeks, every 2 weeks) with longitudinal data analysis.

### Statistical analysis Questionnaire

By means of linear mixed model analyses differences between visitors of the intervention and control group worksite cafeterias will be obtained. Also differences in satisfaction with the worksite cafeteria before and during the intervention will be analyzed with a linear mixed model. Satisfaction with the worksite cafeteria will be subdivided in satisfaction with the products offered, the price of products and the way and order that products are placed. A regression analysis will be obtained to take possible confounding variables into account.

Descriptive statistics of the worksite cafeterias will be used to characterize the intervention and control group at baseline. Moreover, descriptive statistics will be used to identify satisfaction, food choice behavior and subjective health of all participating employees in the pre-test.

Statistical analyses will be conducted using standard statistical computer software (IBM SPSS Statistics 20.0) and MLwiN 2.35 software for mixed models. All statistical tests will be two-tailed and a 5% significance level will be maintained throughout the analyses.

## Discussion

The objective of this study was to develop an intervention (named: “the worksite cafeteria 2.0”) based on nudging and social marketing techniques to make purchase behavior of Dutch employees healthier. Furthermore, the aim was to describe the design of a study to measure the effect of multiple simultaneously executed strategies in “the worksite cafeteria 2.0” on purchasing behavior of visitors in Dutch worksite cafeterias. Thereby answering the research question: What is the effect of a healthier worksite cafeteria based on nudging and social marketing techniques on the purchasing behavior of employees?

To our knowledge there are no studies that made a combination of evidenced based strategies with nudging and social marketing strategies and that are tested in ‘real-life’. Whereby ‘real-life’ means in different real worksites with different catering companies.

We will discuss several strengths of this study. A first strength considering the design is the fact that the effect will be tested in real-life Dutch worksite cafeterias, taking the variety between catering companies and industrial branches into account. This has the advantage over other studies that it gives realistic insight in the effect of the intervention in real-life settings and increasing generalizability, but it also will provide insight in the support for such intervention. By means of organizing this intervention study one gets insight in the amount of effort it takes to convince several companies to implement the strategies, in other words, insight in the amount of support that is needed for continuous implementation.

Second, to choose worksite cafeterias as a target location gives the opportunity to reach many people at their daily routine of visiting the worksite cafeteria. Since people will not have to sign up themselves, probably also people who are not traditionally engaged in health promotion campaigns can be reached. This could be an addition to health interventions reaching mostly only motivated people. Offering a solution for those people not intrinsically motivated would fill a gap.

A third strength of this intervention development and trial is the collaboration with multiple stakeholders like several catering companies, the Netherlands Nutrition Centre, Ministry of Health, Youth on a Healthy Weight (JOGG) and Veneca. By means of involving several catering companies the intervention will be developed and tested in practice. Working with catering companies from the start can tackle the common gap between research and practice, especially in the practical feasibility. The collaboration with Veneca enables the implementation of “the worksite cafeteria 2.0” on a larger scale. The position of Veneca gives them the ability to reach all catering companies and other stakeholders needed when making agreements for contract catering industry.

The last strengths to mention concerning the design is that the effect will be tested with a randomized controlled trial and by using objective data collection, namely sales data. Randomly allocating worksite cafeterias to the intervention group or to the control group is considered the golden standard for determining the efficacy of interventions and objective data are preferred over subjective data.

Finally also the intervention itself has some important strengths. The use of nudging and social marketing strategies is a promising tactic in changing people’s behavior [54, 55]. Just changing the environment has the potential effect of not invoking negative reactions. Furthermore, executing effective strategies simultaneously can have a cumulative effect and could be more effective in a heterogeneous group.

The present study is also subject to some limitations that need to be acknowledged. First, when recruiting worksite cafeterias for the RCT some bias can be expected. Probably companies who are more interested in a healthy lifestyle are more willing to participate. These worksite cafeterias will probably already have a healthier assortment and therefore the effect could possibly be relatively small. Therefore, in recruiting worksites we will put extra effort in including companies with a so-called blue collar workforce. Second, although a minimum of strategies must be executed, some strategies will not be applicable in certain cafeterias which may result in diversity within the interventions tested. For example, offering a smaller portion of a hot meal is not applicable if a cafeteria does not sell hot meals. Non applicable strategies could lead to an intervention less effective and differences in the intervention could make it difficult to interpret the effect. However, also in the control group some strategies would have not be applicable in some worksites. This will reflect the real-life execution and effect of such intervention.

The third limitation is the possibility of missing or false data, as a result of incorrect registration of products at the cash desk. Although the majority of the worksite cafeterias scan most of their products at the cash desk, some products will be registered with buttons. This could lead to incorrect registration of products. A final limitation is that the correct realization of all strategies cannot be controlled by the research team every day. Catering employees will be trained to execute the strategies as correctly as possible and bi-weekly the research team will visit the intervention cafeterias unannounced to check whether the strategies are executed correctly.

In conclusion, this healthy worksite cafeteria intervention is based on a unique combination of nudging and social marketing techniques. It will facilitate employees to purchase healthier products in real-life worksite cafeterias. By developing this intervention with input of employees and in close cooperation with catering and nutrition experts and the most important catering companies in the Netherlands, it has a good chance of long-term implementation.
